# B cells oppose *Mycoplasma* *pneumoniae* vaccine enhanced disease and limit bacterial colonization of the lungs

**DOI:** 10.1038/s41541-022-00556-z

**Published:** 2022-10-31

**Authors:** Tyler D. Gavitt, Arlind B. Mara, Meagan L. Goodridge, Rosemary Grace Ozyck, Emily Reinhardt, Jeremy M. Miller, Morgan Hunte, Edan R. Tulman, Salvatore Frasca Jr, Lawrence K. Silbart, Steven J. Geary, Steven M. Szczepanek

**Affiliations:** 1 Department of Pathobiology and Veterinary Science, University of Connecticut, Storrs, CT 06238 USA; 2 Center of Excellence for Vaccine Research, University of Connecticut, Storrs, CT 06238 USA; 3 US Animal Vaccinology Research Coordination Network, Storrs, CT 06238 USA; 4 Connecticut Veterinary Medical Diagnostic Laboratory, Storrs, CT 06238 USA; 5 Department of Allied of Health Sciences, University of Connecticut, Storrs, CT 06238 USA; 6 Present Address: Sanofi, Meriden, CT 06450 USA

**Keywords:** Protein vaccines, Protein vaccines

## Abstract

Development of an effective vaccine for *Mycoplasma pneumoniae* has been hindered by reports of Vaccine Enhanced Disease (VED) in test subjects vaccinated and challenged in studies conducted in the 1960s. The exact mechanism of disease exacerbation has yet to be fully described, but host immune responses to Lipid-Associated Membrane Proteins (LAMPs) lipoprotein lipid moieties have been implicated. LAMPs-induced exacerbation appears to involve helper T cell recall responses, due in part to their influence on neutrophil recruitment and subsequent inflammatory responses in the lung. Herein, we characterized the functions of host B cell responses to *M. pneumoniae* LAMPs and delipidated-LAMPs (dLAMPs) by conducting passive transfer and B cell depletion studies to assess their contribution to disease exacerbation or protection using a BALB/c mouse model. We found that antibody responses to *M. pneumoniae* LAMPs and dLAMPs differ in magnitude, but not in isotype or subclass. Passive transfer, dLAMP denaturation, and monoclonal antibody studies indicate that antibodies do not cause VED, but do appear to contribute to control of bacterial loads in the lungs. Depletion of B cells prior to LAMPs-vaccination results in significantly enhanced pathology in comparison to B cell competent controls, suggesting a possible regulatory role of B cells distinct from antibody secretion. Taken together, our findings suggest that B cell antibody responses to *M. pneumoniae* contribute to, but are insufficient for protection against challenge on their own, and that other functional properties of B cells are necessary to limit exacerbation of disease in LAMPs-vaccinated mice after infection.

## Introduction

*Mycoplasma pneumoniae* is an atypical bacterial pathogen of humans and a leading cause of community acquired pneumonia (CAP) worldwide^1^. It is estimated that *M. pneumoniae* causes as many as 20% of CAP cases in the US annually, resulting in ~100,000 adult hospitalizations^1^. In some demographics, especially in young children and the elderly, this pathogen can be responsible for up to 40% of CAP cases^2^. *M. pneumoniae* infection has been associated with a number of extrapulmonary manifestations, including neurological and vascular complications which can drastically increase the severity of disease and associated morbidity and mortality^2–4^. *M. pneumoniae* infection is also known to exacerbate other respiratory diseases including asthma, with some reports suggesting that *M. pneumoniae* infection may even predispose individuals to the development of asthma^5–7^. At present, *M. pneumoniae* pneumonia often goes undiagnosed due to a lack of readily available diagnostic tests, as well as commonalities between symptoms of *M. pneumoniae* infection and those of other common respiratory pathogens of humans. Therefore, its true contribution to CAP case burden is likely dramatically underestimated^8^. The atypical biology of *M. pneumoniae* contributes to difficulties in diagnosing and treating infected individuals. *Mycoplasma* species are notoriously fastidious, and laboratory culture is difficult and time consuming^3^, which often leads to an aversion to the use of culture in typical diagnostic approaches. *Mycoplasmas* lack the peptidoglycan cell wall present in most bacteria, which lends inherent resistance to β-lactam class antibiotics that inhibit cell wall synthesis^9,10^. In addition, in recent years there has been a notable increase in the proportion of *Mycoplasma* isolates resistant to macrolide-class antibiotics, a frequent treatment option for *Mycoplasma* infections^11,12^.

*Mycoplasma* are highly adept at evading host immunity. Millions of years of reductive evolution has resulted in a contraction of the genomes of Mycoplasmas and a preservation only of the genes necessary for the survival of the pathogen. This reductive evolution explains the parasitic nature of *Mycoplasma* pathogens, whereby they must attain nutrients from host cells in order to fulfill their life cycle. Due to their lack of a cell wall these bacteria are instead bounded by “triple-layered” cell membranes embedded with numerous lipoproteins. These lipoproteins are recognized as antigens which contribute to immune dysregulation in infected hosts, which allows the bacteria to subvert innate and adaptive immunity to promote its longevity. *Mycoplasma* lipoproteins primarily drive inflammation through their stimulation of pattern recognition receptors in the Toll-like Receptor (TLR) family^13–15^. TLR2/6 and TLR1/2 heterodimer complexes recognize diacylated and triacylated lipoproteins, respectively^16–21^. Uniquely, *Mycoplasma* possess lipoproteins of both classes^14^. TLR ligation has significant effects on T cell differentiation and helper T cell polarization (reviewed by ref.)^22^, and these lipoproteins are significant drivers of T cell responses to *Mycoplasma pneumoniae*.

A vaccine capable of preventing *M. pneumoniae* pneumonia would drastically reduce CAP burden worldwide, improve human health, and decrease burden on the healthcare industry. In addition, prevention of CAP would reduce lost work time and other economic impacts of the disease. Researchers in the 1960’s attempted to develop vaccines for *M. pneumoniae* using formalin-inactivated whole pathogen, but these efforts were stymied by the observation that some vaccinated individuals experienced enhanced clinical symptoms upon subsequent infection in comparison to unvaccinated control subjects^23^. This finding, later termed Vaccine-Enhanced Disease (VED), has been recapitulated in mouse models of *M. pneumoniae* infection, and has stood as a roadblock to successful vaccine development for several decades^24–27^. Prior exposure to live or inactivated *M. pneumoniae* followed by subsequent challenge consistently leads to increased lung pathology characterized by enhanced leukocyte infiltration and perivascular/peribronchiolar cuffing^24,27^. Recently, our group identified *Mycoplasma* Lipid-Associated Membrane Protein (LAMP) lipoprotein lipid moieties of *M. pneumoniae* as the causative agent of disease exacerbation in mice by demonstrating that lipoprotein lipase treated “delipidated“ LAMPs (dLAMPs) did not induce exacerbation upon vaccination and challenge of BALB/c mice, whereas LAMP vaccination and challenge did^27^. Exacerbated *M. pneumoniae* infection appears to be related to the function of adaptive immune cells, as prior exposure to live or killed *M. pneumoniae* or LAMP fraction lipoproteins is required to elicit VED in mouse models^24–27^. CD4 + T cells appear to be major coordinators of the exacerbated inflammatory response through their secretion of pro-inflammatory cytokines and chemokines, which recruit large numbers of neutrophils to the lung and cause significant bystander injury to the tissue^27^. In the interest of further clarifying the roles of adaptive immune cells in this model, we sought to analyze the contributions of B cells to protection from infection with *M. pneumoniae* after vaccination with the LAMP fraction. In contrast to what is seen in other examples of VED, we find that B cell responses against *M. pneumoniae* may actually provide benefit to the host, even when induced by exposure to the LAMP fraction, but these B cell responses appear to be insufficient to elicit protection under the significant inflammatory burden of the maladaptive T cell response.

## Results

### Characterization of murine antibody responses to LAMPs and dLAMPs

**Fig. 1:**
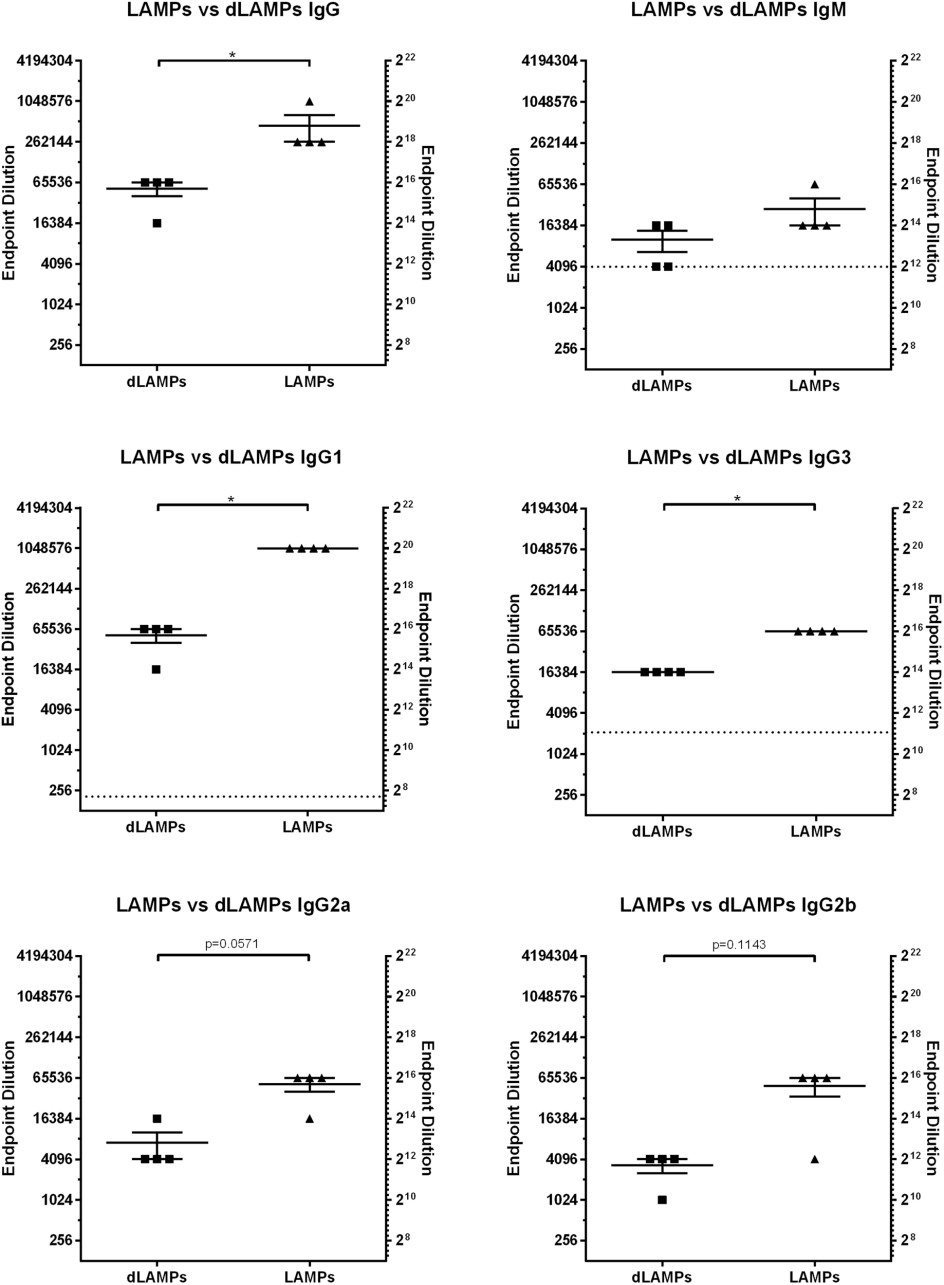
Serum antibody responses to Lipid-associated Membrane Proteins (LAMPs) or delipidated Lipid-associated Membrane Proteins (dLAMPs) in vaccinated mice. Endpoint titers are displayed as the reciprocal of the final dilution at which signal was at least three times greater than the assay background. Data were analyzed by Mann–Whitney Test. Data were considered significant for *p* < 0.05. Data without significance marking is to be considered not significantly different. Dotted line indicates the lower limit of detection of the assay.

Mice were vaccinated on day 0 and boosted 21 days later via intraperitoneal (IP) injection with LAMPs, dLAMPs, or physiological saline (sham). On day 42 all animals were euthanized for sample collection. Serum antigen-specific antibody isotypes and subclasses were analyzed by indirect ELISA, with endpoint titers reported as the inverse of the endpoint dilution. Investigation of the induced antibody isotypes and subclasses revealed that antibody responses to both vaccine formulations differed in magnitude, but not in isotype or subclass distribution. Overall, the relative magnitude of *M. pneumoniae*-specific serum IgA and IgE were low in comparison to IgG (Fig. 1, Supplementary Fig. 1). Vaccination with LAMPs and dLAMPs induced robust IgG responses by day 42, with LAMP-vaccinated mice demonstrating significantly higher serum IgG titers in comparison to dLAMP-vaccinated mice. LAMP-vaccination also induced significantly higher *M. pneumoniae*-specific IgG1 and IgG3 titers in comparison to dLAMP vaccination (Fig. 1). Endpoint titers of IgM, IgG2a, and IgG2b were not significantly different between LAMP- and dLAMP-vaccinated mice, but the trend of higher responses to LAMPs than dLAMPs was maintained. These same distributions of antibody prevalence were mirrored in the Bronchoalveolar Lavage Fluid (BALF), with IgG1 responses dominant (Supplementary Fig. 2). These findings suggest that both LAMP- and dLAMP-vaccination induces robust antibody responses toward *M. pneumoniae*-derived antigens. However, while the magnitude of the antibody response was different between the two groups, the removal of the lipoprotein lipid moieties in the dLAMP formulation does not appear to have altered the isotype or subclass distribution of the antibody response.

### Passive transfer of vaccine-induced antibody to naïve mice

**Fig. 2:**
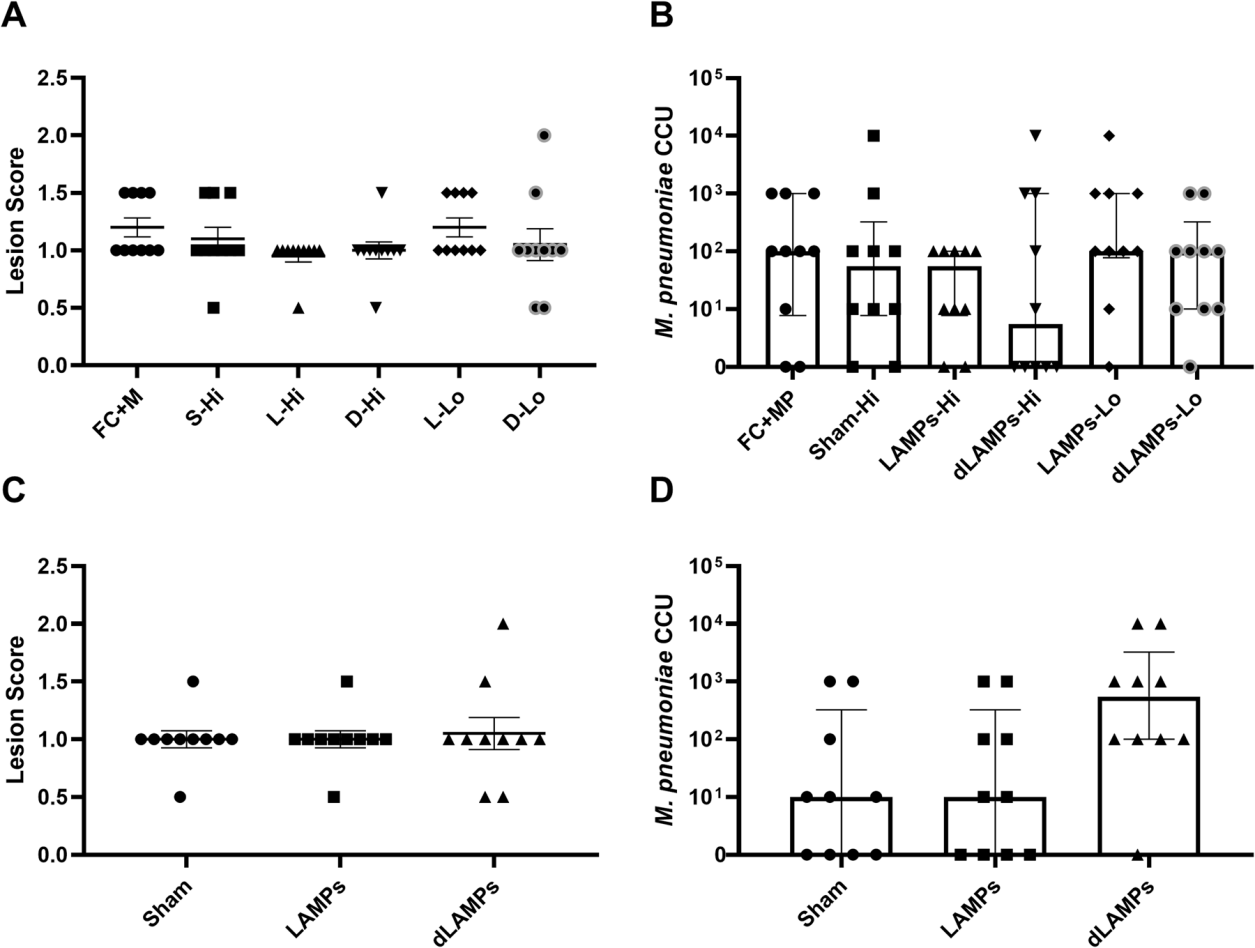
Passive transfer of hyper-immune sera to naïve mice. **A** Histopathologic scoring of lungs from mice intranasally inoculated with Lipid-associated Membrane Protein-induced (LAMP), delipidated Lipid-associated Membrane Protein-induced (dLAMP) or naïve-mouse (sham) serum at high (Hi) or low (Lo) concentration and challenged with *M. pneumoniae*. Data were compared by Kruskal–Wallis test with Dunn’s post-hoc test used for multiple comparisons. **B** Bacterial recoveries from the lungs of mice intranasally inoculated with vaccine-induced or naïve-mouse (Sham) serum at high or low concentration and challenged with *M. pneumoniae*. Data are displayed as median with interquartile range, and were compared by Kruskal–Wallis test with Dunn’s post-hoc test used for multiple comparisons. **C** Histopathologic scoring of lungs from mice intraperitoneally inoculated with vaccine-induced or naïve-mouse (sham) serum and challenged with *M. pneumoniae*. Data were compared by Kruskal–Wallis test with Dunn’s post-hoc test used for multiple comparisons. **D** Bacterial recoveries from the lungs of mice intraperitoneally inoculated with vaccine-induced or naïve-mouse (Sham) serum and challenged with *M. pneumoniae*. Data are displayed as median with interquartile range, and were compared by Kruskal–Wallis test with Dunn’s post-hoc test used for multiple comparisons.

In order to investigate the ability of LAMP or dLAMP-induced antibodies to cause VED or protect from infection, passive transfer studies were conducted using either IP or intranasal (IN) transfer of polyclonal, hyperimmune serum induced by LAMPs-, dLAMPs-, or saline-sham vaccination. For IN passive transfer, BALB/c mice were inoculated with 50 μL total volume containing 12.5 μL of pooled donor serum [HI groups] or 1.25 μL of pooled donor serum [LO groups], with the remaining volume comprised of Fortified Commercial (FC) media containing 5 × 10^7^ CFU *M. pneumoniae* strain PI1428. Mice that received IP passive transfer got 150 μl of pooled donor serum in 100 μl of saline, with a separate IN challenge. Four days post-infection, animals were euthanized to assess bacterial recoveries and histopathologic analysis of lung tissues. Mice that received hyperimmune serum transfer from LAMP- or dLAMP-vaccinated mice were not different with regard to lung lesions in comparison to animals receiving serum from saline-vaccinated control mice, regardless of the route of administration (Fig. 2A, C). Recovered bacteria, as measured by color-changing-units (CCU), were also not significantly different between animals receiving hyperimmune or naïve-mouse serum transfer, regardless of the route of administration (Fig. 2B, D). These data suggest that, even when present in high titers at the surface of the lung or in the systemic circulation, the presence of hyperimmune anti-sera alone is not sufficient to recapitulate VED nor provide protection from infection, as lesion scores and bacterial burdens were not different in animals provided passive transfer of *M. pneumoniae*-specific antibody.

### Passive transfer of α-P1 monoclonal antibody to naïve mice

**Fig. 3:**
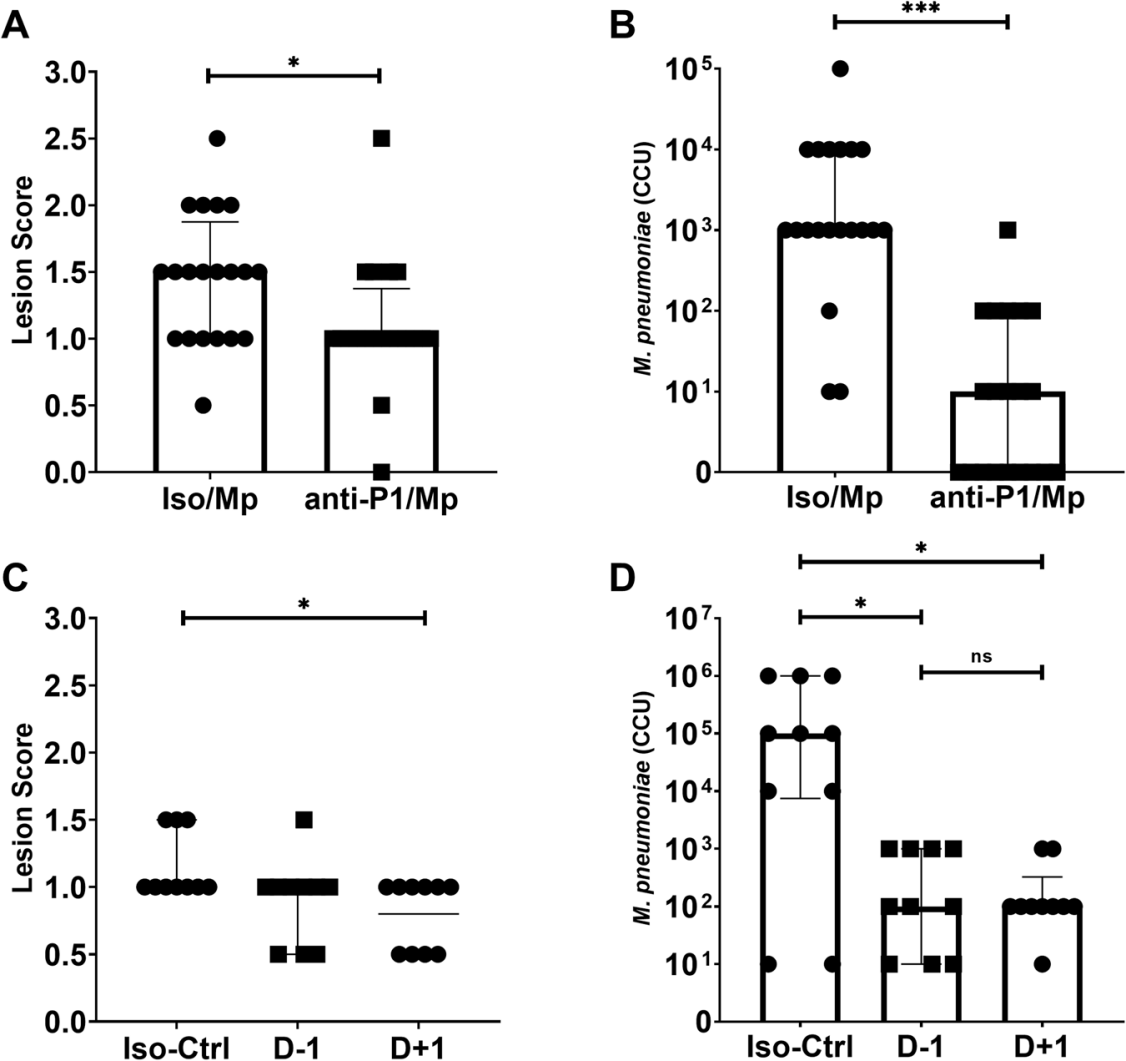
Passive transfer of anti-P1 monoclonal antibody to naïve mice. **A** Histopathologic scoring of lungs from mice intranasally inoculated with a-P1 monoclonal antibody and challenged with *M. pneumoniae*. Data points were compared by Mann–Whitney Test. **B** Bacterial recoveries from the lungs of mice intranasally inoculated with a-P1 monoclonal antibody one day before challenge (D-1) or one day after challenge (D + 1) with *M. pneumoniae*. Data are displayed as median with interquartile range. Data points were compared by Mann–Whitney Test. **C** Histopathologic scoring of lungs from mice intraperitoneally inoculated with a-P1 monoclonal antibody and challenged with *M. pneumoniae*. Data were compared by Kruskal–Wallis test with Dunn’s post-hoc test used for multiple comparisons. **D** Bacterial recoveries from the lungs of mice intraperitoneally inoculated with a-P1 monoclonal antibody and challenged with *M. pneumoniae*. Data are median with interquartile range. Data compared by Kruskal–Wallis test with Dunn’s post-hoc test used for multiple comparisons. Differences for all comparisons were considered significant for *p* < 0.05 (*p < 0.05, **p < 0.01, ***p < 0.001).

As hyperimmune sera passive transfer did not alter disease outcome in our model, we sought to identify whether monoclonal antibodies to the primary *M. pneumoniae* attachment protein P1 could elicit a protective phenotype in mice. In previous work, our research group has described a monoclonal antibody specific to the *M. pneumoniae* P1 adhesin, a surface protein critical to *M. pneumoniae* adhesion and gliding motility and built on a murine IgG1 backbone^28^. We administered this antibody to mice in passive transfer experiments similar in design to the serum passive transfer studies described above. In comparison to isotype controls, animals that receiving α-P1 IN had significantly reduced lung lesion scores (Fig. 3A) and significantly lower bacterial recoveries (Fig. 3B) at 4 days post-challenge. This study was repeated using IP passive transfer of α-P1 mAb one day pre-challenge or one day post-challenge (D−1 and D + 1, respectively) to verify these results. Animals who received IP passive transfer of α-P1 mAb on D−1 had similar lung lesion scores (Fig. 3C), but significantly lower bacterial recoveries (Fig. 3D) in comparison to isotype control animals. Animals who received IP passive transfer of α-P1 on D + 1 had significantly lower lung lesion scores and bacterial recoveries than isotype control animals. These data suggest that in naïve mice, passive transfer of high dose monoclonal antibody directed at a critical *M. pneumoniae* attachment protein provided protection against high dose challenge with live bacteria. These findings suggest a possible role for *M. pneumoniae*-specific antibody responses in controlling bacterial populations in the lung after challenge.

### Vaccination with native and denatured dLAMPs

**Fig. 4:**
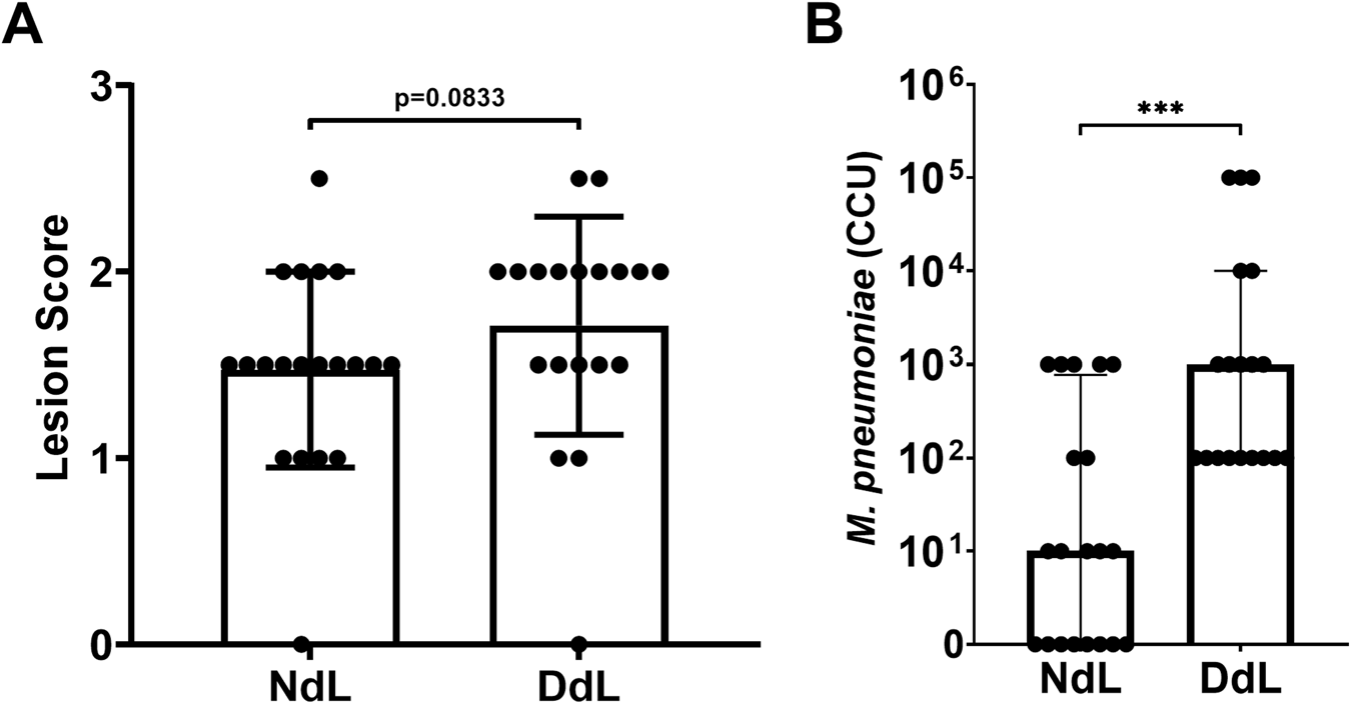
Vaccination with native or denatured dLAMPs. **A** Histopathology scoring of lungs from mice intraperitoneally vaccinated with native conformation (NdL) or denatured (DdL) dLAMPs and then intranasally challenged with *M. pneumoniae*. Data were compared by Mann–Whitney Test and are displayed as median with interquartile range. **B** Bacterial recovery from lungs from mice intraperitoneally inoculated with NdL or DdL and intranasally challenged with *M. pneumoniae*. Data points were compared by Mann–Whitney Test, and differences were considered significant for *p* < 0.05 (***p < 0.001).

In contrast to the exacerbated disease induced by vaccination with the LAMP fraction, previous vaccination-and-challenge studies indicate that vaccination with the dLAMP fraction significantly reduces bacterial burden in the lungs^27^. To further investigate whether antibody responses contribute to the reduction in lesion severity seen in the dLAMP vaccination-and-challenge model^14^, we sought to ablate conformational epitopes within the dLAMP fraction via high-temperature protein denaturation to analyze the roles of *M. pneumoniae*-specific antibodies. Lung histopathologic lesion scores were not significantly different after challenge between the denatured (DdL) and non-denatured (native, NdL) dLAMPs vaccine groups (*p* = 0.083), but overall lung pathology trended higher in the group receiving the DdL dLAMPs (Fig. 4A). Mice vaccinated with native dLAMPs (NdL) had significantly fewer bacteria recovered from the lungs in comparison to those vaccinated with the heat-DdL dLAMPs fraction (Fig. 4B). Notably, IgG responses were measurable in animals receiving the NdL formulation, but such responses were also evident in animals receiving DdL dLAMPs, suggesting that the DdL proteins retained immunogenicity, but were ineffective at reducing bacterial colonization (Supplementary Fig. 3). Thus, the increased bacterial recoveries in the group vaccinated with DdL dLAMPs suggests that antibody responses play an important role in controlling infection, and modification of discontinuous or conformational epitopes, for which B cells reportedly have a preference^29,30^, potentially compromises protection.

### B cell depletion in LAMPs-vaccinated mice

**Fig. 5:**
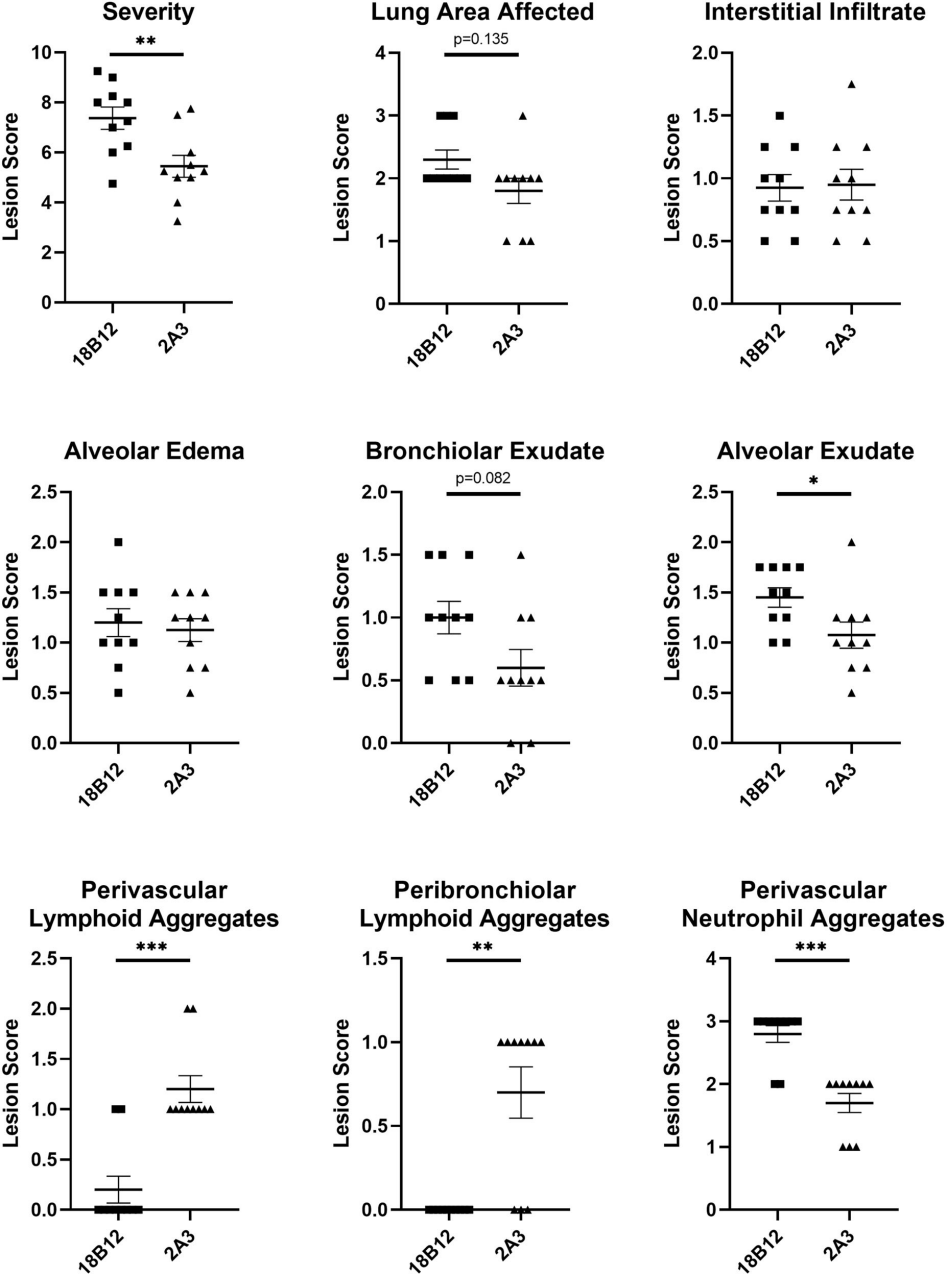
Pathologic effects of B cell depletion on LAMP vaccination and infection. Histologic outcomes of mice vaccinated with *M. pneumoniae* LAMPs and pre-treated with the B cell depleting antibody 18B12.1 or the isotype control antibody 2A3. Severity score is the overall sum of interstitial infiltrate, alveolar exudate, alveolar edema, bronchiolar exudate, and perivascular neutrophil aggregate scores for individual animals. Data were analyzed using the Mann–Whitney test, displayed as mean plus and minus standard error of the mean, and differences were considered significant for *p* < 0.05 (*p < 0.05, **p < 0.01, ***p < 0.001).

To determine the contribution of LAMP-induced B cell responses to the antibody responses against *M. pneumoniae* infection and VED, we depleted B cells prior to and during LAMP vaccination using a commercially available anti-CD20 monoclonal antibody (18B12.1, a modified 18B12 clone^31^ utilizing the IgG2a constant region). *M. pneumoniae*-specific IgG titers were significantly reduced in animals that received 18B12.1 treatment in comparison to 2A3 (isotype) treated controls (Supplementary Fig. 4). After challenge, bacterial recoveries from 18B12.1 and 2A3 treated animals were not significantly different (Supplementary Fig. 5). Notably, upon examination of histologic sections of lungs from B cell-depleted animals, the typical perivascular-peribronchiolar lymphocytic infiltrates were abnormal. The near absence of perivascular and peribronchiolar lymphoid aggregations in 18B12.1-treated, B cell-depleted mice suggests that either B cells represent the majority cell population in these lesions, or that the absence of B cells somehow results in an additional loss of T cells from these lesions. When perivascular/peribronchial lesions were present in 18B12.1-treated mice, they were considerably less severe than in 2A3 isotype-treated animals or sham vaccinated animals, but overall, the severity of disease in the lungs of 18B12.1-treated mice appeared markedly increased. Overall, there appeared to be a greater degree of consolidation present in 18B12.1-treated animals, with increased lung involvement, despite the lack of characteristic lesions. The atypical lung lesion characteristics in 18B12.1-treated mice was most likely attributable to the B cell depletion in those animals, and the scoring system based on perivascular and peribronchiolar lymphoid aggregations utilized in previous studies was insufficient to appropriately gauge differences in disease severity between LAMP vaccinated and B cell-depleted or isotype-treated animals. A new lesion scoring system was developed and utilized to specifically evaluate LAMP vaccinated B cell-depleted animals relative to LAMP vaccinated isotype control animals. In comparison to 2A3-treated control mice, 18B12.1-treated mice had higher aggregate severity score and lung area affected trended larger (*p* = 0.135) (Fig. 5). Alveolar exudate was significantly increased and bronchiolar exudate trended toward an increase (*p* = 0.082) in LAMP vaccinated B cell-depleted animals. Of note, depletion of B cells during LAMP vaccination resulted in a dramatic increase in perivascular neutrophil accumulation after infection.

### Cellular composition of lesions in LAMP vaccinated and B cell-depleted mice

**Fig. 6:**
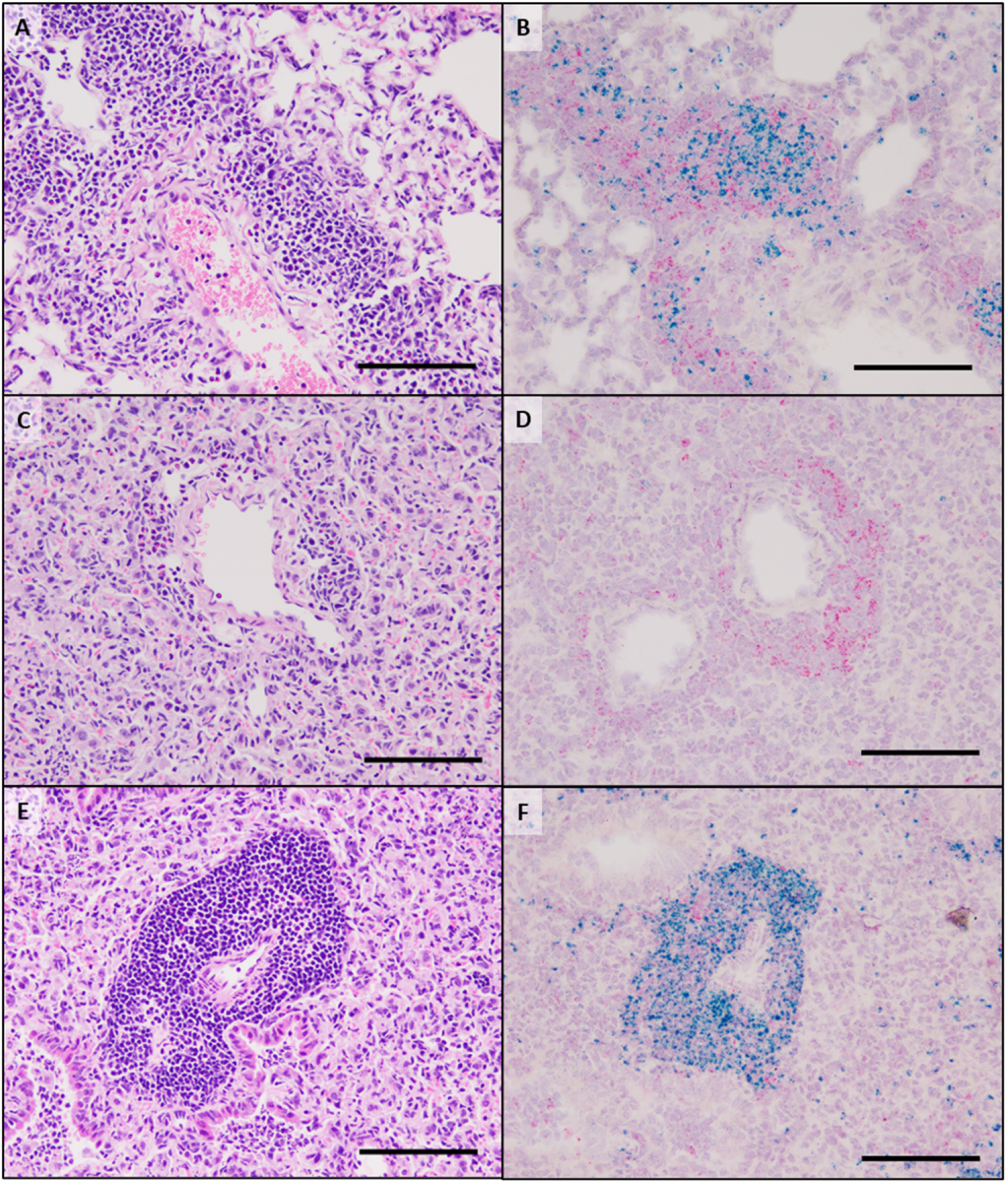
In situ hybridization of B cells and T_h_ cells in LAMP vaccinated and infected mice after B cell depletion. H&E staining (**A**, **C**, **E**) and RNAscope in situ hybridization (**B**, **D**, **F**) of lung sections from mice vaccinated with the LAMP fraction and treated with the isotype control antibody 2A3 (**A**, **B**) or a B cell depleting antibody 18B12.1 (**C**, **D**) prior to challenge with live *M. pneumoniae*. Sham vaccinated and challenged animals were included as reference controls (**E**, **F**). Green chromogen deposits correspond to CD19 mRNA, red chromogen deposits correspond to CD4 mRNA. **A**, **B** A large pulmonary vessel representing the typical cellularity and distribution of lymphocytes forming a perivascular cuff. Cells with CD19 mRNA form multifocal aggregates whereas cells with CD4 mRNA are scattered around the vessel. Low numbers of neutrophils are within the lumen of adjacent alveoli. **C**, **D** In a B cell-depleted mouse, the alveolar septa are markedly expanded by high numbers of macrophages, which in many regions are accompanied by a severe neutrophilic exudate (not imaged). Within this area there are relatively few lymphocytes forming a small cuff around a pulmonary vessel. The majority of these cells express chromogen for CD4 mRNA. **E**, **F** In a region of similar severity of pneumonic lesions in a Sham vaccinated mouse, there is a robust perivascular cuff composed of 7–20 cell layers of lymphocytes. With RNAscope in situ hybridization there is a distribution of high numbers of cells with CD19 mRNA and lesser numbers of cells with CD4 mRNA. All scale bars are equivalent to 100 μm.

RNAScope in situ hybridization was utilized to specifically identify CD19 + B cells and CD4 + T cells in formalin-fixed, paraffin-embedded lung tissues from LAMP vaccinated mice receiving either 18B12.1 or 2A3. Perivascular and peribronchiolar lesions present in LAMP-vaccinated 2A3-isotype-treated mice (i.e., “typical VED”) contained mixed populations of CD19+ and CD4 + cells, with a predominance of CD19 + populations. In most cases these cells were fully circumferential to the adjacent bronchiole or vessel, though CD19 + B cell populations in these lesions were more focal in comparison to CD4 + cell populations (Fig. 6A, B). The focal nature of these B cells likely explains, at least in part, the atypical appearance of the lesions in B cell-depleted mice, as the more diffuse nature of lymphocytes in the periphery of the airways and vessels prevented visualization of these cells when observed under light microscopy with H&E staining. Analysis of RNAscope-ISH slides from LAMP-vaccinated 18B12.1-treated mice revealed the near complete absence of CD19 + cells, further confirming the success of the depletion (Fig. 6C, D). However, CD4 + cell populations were still visible in the areas surrounding bronchioles and small vessels in the areas of the mouse lung experiencing the most severe inflammatory responses post-challenge. These CD4 + T cell populations were often present in the entire periphery of the affected airway or vessel. Also of note, lesions in sham vaccinated and challenged mice showed lesions that consisted mostly of B cells (Fig. 6E, F), indicating that B cells are responding to the presence of the pathogen and accumulating in the lungs as early as 4 days after infection.

## Discussion

Many vaccines aim to elicit high-titer binding and/or neutralizing antibody responses to prevent pathogen interaction with host tissues, and for many pathogens these antibody responses are protective. In rare circumstances vaccines can elicit non-protective immune responses which predispose the host to more severe disease upon exposure to the pathogen, a phenotype commonly referred to as VED. Vaccines against Dengue Virus^32–34^, Respiratory Syncytial Virus^35–39^, and atypical Measles^40–43^ are commonly associated with VED, and their mechanism has been identified as Antibody-Dependent Enhancement (ADE) of disease. The current study investigated whether *M. pneumoniae* VED shares this mechanism.

Recent work from our lab has shown that *M. pneumoniae* LAMPs induce a heightened inflammatory response after challenge, resulting in VED. Conversely, de-lipidation of LAMP proteins (dLAMPs) ablates VED^27^. Vaccination with dLAMPs significantly reduces the post-challenge inflammatory response, supporting the notion that lipoprotein lipid moieties are potent mediators of *Mycoplasma* immune dysregulation. To assess the role of antibodies in VED, we characterized antibody titers, isotype and subclass distribution in responses to vaccination with *M. pneumoniae* LAMPs or dLAMPs. Each vaccine preparation was administered without adjuvant to avoid artificially biasing the ensuing immune response. Surprisingly, the antibody titer in both groups was very high, reaching titers of >65,000 in dLAMP vaccinated mice and >262,000 in LAMP vaccinated mice (Fig. 1). The notable difference in titers is ostensibly due to the intrinsic adjuvant activity of the lipoprotein lipid moieties due to TLR ligation, since the protein content of each vaccine was otherwise identical. We hypothesized that there would be significant differences in antibody isotype and possibly subclass distribution due to the aforementioned TLR ligation^44,45^, but surprisingly this was not the case. In both groups, serum and bronchoalveolar lavage (BAL) fluid antibody responses were overwhelmingly IgG1 dominant, with only a modest IgM response and negligible titers of IgA and IgE. (Fig. 1, Supplementary Fig. 1).

To determine if antibody responses directly contribute to VED, we conducted passive transfer experiments using hyperimmune immune serum from LAMPs or dLAMPs vaccinated mice followed by *M. pneumoniae* challenge. Surprising, neither group exhibited exacerbated disease following challenge (Fig. 2), allowing us to rule out antibodies as a cause of VED. Curiously, the transferred high-titer antibody response was only moderately effective in reducing bacterial colonization of the lung, leading us to hypothesize that LAMPs may be inducing an irrelevant serum antibody response, as has been suggested with other Mycoplasmas^46^. To address this hypothesis, we passively transferred a monoclonal antibody directed against the P1 adhesin, which interferes with bacterial attachment (Fig. 2). In these mice the anti-P1 antibodies mediated partial protection, ostensibly due to a reduction in bacterial attachment or motility (Fig. 3). As anticipated, vaccination with heat-DdL dLAMPs resulted in a loss of protection as evidenced by significant increases in bacterial burden in the lungs (Fig. 4). Taken together, our results are similar to the work of others and demonstrate that antibody responses to LAMPs do not contribute to VED, but do play an important role in reducing bacterial lung colonization^47–54^.

As B cells are typically present in the characteristic perivascular and peribronchiolar leukocytic aggregates observed during *M. pneumoniae* infection, we sought to understand their role in VED. We hypothesized that lesion severity would be lessened in the absence of B cells, given the presence of lymphocyte accumulations during the exacerbated inflammatory response. Surprisingly, B cell depletion actually led to a significant increase in the overall severity of pneumonia, an increase in the area of the lung affected, and increased neutrophilic accumulation in the lungs (Fig. 5). Thus, it appears that B cells in the lung parenchyma after challenge with *M. pneumoniae* may be playing an immunoregulatory role, as has been observed in other disease states^55–58^. To address this hypothesis, RNAScope in situ hybridization was used to assess the cellular composition and organization of the lymphocytic infiltrates. In sham vaccinated and challenged animals, these accumulations were overwhelmingly comprised of CD19 + B cells (Fig. 6E,F), indicating that *M. pneumoniae* infection in naïve mice induces a robust B cell responses by day 4 post-infection. In LAMP-vaccinated and challenged animals, these regions increase in size and density and contain mixed populations of disorganized regions of CD4 + T cells and CD19 + B cells. Of note, these accumulations were more organized in dLAMP-vaccinated and challenged animals, with well-demarcated B and T cell zones reminiscent of structures seen in secondary lymphoid tissues (Supplementary Fig. 6).

Having shown that antibody responses to *M. pneumoniae* are insufficient to recapitulate VED after passive transfer and subsequent challenge of naïve BALB/c mice, we conclude that *M. pneumoniae* VED is not an example of ADE. Hyperimmune serum from LAMPs and dLAMPs-vaccinated mice was also insufficient to confer protection upon challenge, a finding in keeping with previous studies^50^. Thus, we reasoned that B cells may contribute to controlling *M. pneumoniae* infection after vaccination and challenge via other mechanisms. This hypothesis was further supported by the observed increases in lung pathology when B cells were depleted. Thus, dysregulated T cell responses may play a critical role in VED, in part due to Th17 responses driven by LAMP ligation of TLR2-containing PRR complexes and the resulting diminution of Treg responses^59^. This hypothesis is supported by a recent report demonstrating that the specific depletion of CD4 + T cells in an *M. pneumoniae* model resulted in a reduction in neutrophilic infiltration to the lungs and proposed a role for CD4 + T cells of type 1 and/or type 17 in the recruitment of these cells to the mouse lung^60^. In addition, investigations of CD4 + T cell behavior in mouse models of the related pathogen *M. pulmonis* revealed a similar decrease in lung pathology after depletion of CD4 + T cells^61^, further implicating Th cells in the dysregulated response. Exposure to *M. pneumoniae* antigen has been reported to induce the expression of inflammatory cytokines associated with the Th17 lineage, including IL-17A and IL-23^26,62–65^, and IL-17A has been shown to exacerbate lung pathology in mice infected with *Mycoplasma pulmonis*^66^. The Th17/Treg axis is dynamic, and heavily influenced by anti-inflammatory cytokines during T cell exposure to antigen. Regulatory B cells may counteract the effects of these inflammatory T cell responses after vaccination with *M. pneumoniae* antigens upon secretion of anti-inflammatory cytokines, including IL-10 and TGF-B^67^. The increased Th17 activity observed in LAMP vaccinated mice is well correlated with a concomitant increase in neutrophilia seen in the lungs (Fig. 5), in keeping with the observation that IL-17A contributes to the secretion of the neutrophil chemotactic factor IL-8^68^ (KC in mice) and is a major driver of neutrophil migration and activation^63,69–71^. B cell derived anti-inflammatory cytokines may temper inflammatory responses to *M. pneumoniae* antigens by inducing the formation of regulatory T cells which help control overexuberant inflammatory responses during infection^72^. Indeed, T cell derived IL-10 has been shown to diminish IL-17A production in response to crude lysate preps containing *M. pneumoniae* antigen^64^, and it may be possible that B cell derived IL-10 could play a similar function. Interleukin-10 levels in the lungs of *M. pneumoniae* infected mice rise sharply after challenge^73^. An early IL-10 response may partially restrict the inflammatory response driven by IL-17A and its downstream effects, but this requires further investigation. Identification of the cells that produce this IL-10 is also necessary to fully understand the role of this cytokine in the response to *M. pneumoniae* infection and VED. We postulate that the absence of cytokine-producing B cells after B cell depletion resulted in a more robust Th17 responses after challenge and the subsequent increase in pulmonary inflammation.

Safety concerns regarding VED have prevented the development of successful vaccines against *M. pneumoniae*, despite clear necessity. We have demonstrated that, in contrast to many other instances of VED described in the literature, *M. pneumoniae* VED is not an example of ADE. Further work investigating site-specific B cell responses in the airways, as well as antibody-independent B cell behaviors including cytokine secretion will help to further clarify the true functions of these cells in the context of *M. pneumoniae* infection. The extensive immune dysregulation induced by *M. pneumoniae* lipoproteins clearly demonstrates that they cannot be safely used as vaccine antigens in their native states, as the presence of lipoprotein lipid moieties will always risk predisposing a vaccine recipient to VED. Rather, *M. pneumoniae* vaccines should aim to elicit high tighter antibody responses against specific antigens of the pathogen, including those known to mediate binding and adhesion, including P1, P30, and P116, though discrete protective antigens remain to be fully determined. *Mycoplasma* pathogens represent clear candidates for rationally designed vaccines due to their extensive immune dysregulation capabilities. An increased understanding of *Mycoplasma* immune dysregulation, lipoprotein-induced VED, and the identification of protective antigens will move the field closer to the development of safe and efficacious vaccines for this important respiratory pathogen.

## Methods

### Ethics statement

All animal studies were approved by the University of Connecticut Institutional Animal Care and Use Committee under protocol #A20-044. All infection studies and use of recombinant proteins were approved by the University of Connecticut Institutional Biosafety Committee under registration #926.

### Mice

All studies utilized 8-week-old female BALB/c mice purchased from Jackson Laboratories (Jackson Laboratories, Bar Harbor ME). In all vaccination and challenge experiments, *n* = 10 mice were used per group, with the exception of the DdL dLAMPs study in which *n* = 30 mice were used per group. Prior to all studies, mice were allowed to acclimate to their new housing environment for 1 week. Mice were housed in the vivarium facility at the University of Connecticut. Food and water were provided ad libitum with animal care coordinated by Animal Care Services of the University of Connecticut.*Preparation of Vaccine Formulations.*

*M. pneumoniae* LAMPs^27^ were extracted utilizing TX-114 partitioning using a slightly modified protocol from the one established by Bordier^74^. *M. pneumoniae* PI1428 cells were cultured in T175 cell culture flasks at 37 °C in complete FC medium until mid-log phase as determined by acid-mediated shift of phenol red dye from red to orange. Adherent bacterial cells were scraped onto the medium then pelleted by centrifugation. The pellet was washed with PBS then solubilized in 5 mL of TS-EDTA buffer (20 mM Tris, 150 mM NaCl, 5 mM EDTA pH 7.6) containing 1 mM PMSF (protease inhibitor) and 2% (v/v) TX-114. The solution was rocked for 2 h at 4 °C, followed by centrifugation at 10,000 × *g* at 4 °C for 10 min to pellet the insoluble phase. The soluble phase was transferred to a new tube and incubated at 37 °C until the solution became cloudy (indicating condensation of detergent micelles), then centrifuged for 15 min at room temperature to separate the detergent and aqueous phases which were then aliquoted into new tubes. Appropriate amounts of TS-EDTA buffer and TX-114 detergent were added to the tubes containing the insoluble, aqueous and detergent fractions to reach a 2% TX-114 solution, and the phase partitioning was repeated twice to clean the fractions. Precipitated *M. pneumoniae* TX-114 fractions (Insoluble: Ins, Aqueous: Aq, and Detergent: LAMPs) were treated with 2500 units of Lipoprotein Lipase from *Burkholderia* sp. (EC 3.1.1.34; Sigma Aldrich, St. Louis, MO) per 2 μg of protein, for 48 h at 37 °C with shaking at 250 RPM on an orbital shaker to generate the dLAMPs. The efficiency of lipoprotein lipase treatment was assessed via a Macrophage Inflammatory Bioassay. Briefly, murine J774A.1 macrophages were stimulated for 6 h with 20 μg of protein from each TX-114 fraction and its delipidated counterpart or 15 μg of LPS as a positive control for macrophage activation. Supernatant TNF-α levels were measured using a commercial murine TNF-α sandwich ELISA (Biolegend, San Diego, CA) according to the manufacturer’s instructions.

### Bacterial strains and growth conditions

*M. pneumoniae* strain PI1428 was utilized for all aspects of this study^14^. For infection studies, frozen 50 µL aliquots of low passage (P13) *M. pneumoniae* PI1428 were thawed and resuspended in 10 mL of complete FC medium (20% heat inactivated horse serum, 5% yeast extract). Cultures were incubated at 37 °C with orbital shaking at 120 RPM. After 5 h, optical density at 620 nm (OD_620_) was used to estimate colony forming units (CFU) counts per mL of culture. Furthermore, color changing unit (CCU) measurements were conducted using tenfold 8 serial dilutions to validate spectrophotometric estimations. Samples were then centrifuged at 2000 × *g* for 10 min at 4 °C, the supernatant decanted, and the pellet suspended to the desired concentration in fresh complete FC medium.

### Vaccination of animals and collection of samples

Thirty 8-week-old female BALB/c mice (*n* = 10 per group) were vaccinated via IP injection on a prime-boost schedule on days 0 and 21. Each group of animals received either 250 μL containing 50 μg *M. pneumoniae* LAMPs or dLAMPs resuspended in sterile physiological saline, or a sham solution of 250 μL physiological saline. Animals were monitored for 48 h after each injection for clinical signs. Twenty-one days post-boost, all animals were euthanized via cervical dislocation for blood collection and BAL. Blood was collected via cardiac puncture using a 1 mL syringe and a 3/4” × 27 G needle, placed into 1.5 mL tubes, and allowed to clot for 30 min at room temperature, at which point it was centrifuged at 1000 × *g* for 5 min. The serum was removed and stored in 1.5 mL tubes until assayed. Bronchoalveolar lavage was conducted by washing each mouse’s entire lung once with 1 mL sterile ice-cold PBS. All samples were then stored at 4 °C until use.

### Characterization of the antibody response to *M.pneumoniae* LAMPs and dLAMPs by enzyme-linked immunosorbent assay

Serum and BALF antibody responses were evaluated by Enzyme-linked Immunosorbent Assay (ELISA). Nunclon Polysorp plates (Thermo Fisher Scientific, Waltham MA) were coated overnight at 4 °C with 100 mL carb/bicarb buffer containing 5 ug/mL *M. pneumoniae* lysate. Prior to the loading of samples, plates were washed 3× with PBST containing 0.05% Tween-20 and blocked for 1 h with 100 uL of 2 ug/mL bovine serum albumin diluted in PBST. For serum samples, endpoint titers were conducted, and fourfold dilutions of serum were created by dilution of the sample into PBST. Endpoint titers were conducted only when it was evident in preliminary tests that antibody responses were present. If sufficient signal to detect antibody was not evident at a 1:100 dilution in PBST, endpoint titers were not conducted and the 1:100 dilution ELISA data was reported instead. Prior to loading samples, plates were washed 3× with PBST, and 100 mL diluted sample was loaded and incubated for 1 h at room temperature. Sample dilutions utilized for the analysis of BALF antibodies were determined empirically, as follows: IgG 1:1,000, IgG1 1:500, all other antibodies 1:100. Plates were then washed 3× as previously described. 100 mL of 1:4000 HRP-conjugated secondary antibody diluted in PBST was loaded and incubated 1 h at room temperature, per manufacturer recommendations. Plates were washed 3× with PBST. 100 mL TMB Substrate Solution was added to each well and plates were incubated in the dark for 5 min, at which point the development was stopped with 100 mL TMB-STOP Solution. The plate was then immediately read at 450 nm on a Cytation 5 plate reader. Endpoint titers were reported as the reciprocal of the final dilution at which well signal exceeded three times the average background signal of the assay.

### Intranasal passive transfer and challenge of BALB/c mice

Sixty 8-week-old Female BALB/cJ mice were purchased from Jackson Laboratories (Jackson Laboratories, Bar Harbor ME). Mice were inoculated intranasally with 50 μL of liquid containing both serum and live *M. pneumoniae* bacteria. The formulations were as follows: For “High” serum, 12.5 μL of LAMPs-, dLAMPs-, or Saline-Sham-induced serum plus 37.5 μL FC media containing 5 × 10^7^ CFU *M. pneumoniae* in PI1428. For “Low” serum, 1.25 μL of LAMPs- or dLAMPs-induced serum plus 48.75 μL FC media containing 5 × 10^7^ CFU *M. pneumoniae* PI1428. Negative control animals received 50 μL containing 5 × 10^7^ CFU *M. pneumoniae*. Serum used for this study was pooled from five mice that had been previously vaccinated. Four days post-inoculation, animals were euthanized by cervical dislocation under isoflurane anesthesia for sample collection. The lower right portion of each mouse’s right lung was removed for bacterial recoveries prior to the complete removal of the remaining portions of the lung for histopathological analysis. Recovery samples were stored in FC media on ice until processing, and lungs were stored in 10% neutral buffered formalin until submission. Recoveries were processed as follows: Lung sections in FC media were vortexed at full speed for 2 min, incubated at 37 °C for 3 h, and the liquid portion of each recovery was then filtered through a 0.45 μm syringe-driven filter. This filtrate was then placed in the top well of a row of a 96 well tissue culture plate, and tenfold serial dilutions were done in FC media. After 28 days, the maximum dilution at which a yellow color-change was detectable was recorded as the CCU value for each animal. CCU values were analyzed as non-parametric data using a Kruskal–Wallis test. For histologic analysis of lungs, full lungs were fixed in 10% NBF for 48 h on a shaker table at room temperature, trimmed, and submitted to the Connecticut Medical Diagnostic Laboratory (CVMDL) at the University of Connecticut for embedding and slide preparation. Slides were cut and stained with hematoxylin and eosin (H&E) by CVMDL and analyzed under a light microscope. Slides were scored in a blinded fashion by an individual with experience scoring lesions seen in this model. Perivascular and peribronchiolar lymphocytic infiltration was graded using the following system; 0—no lesions, 1—mild lesions, 2—moderate lesions, 3—marked lesions, 4—severe lesions. Half-step intervals (i.e., + 0.5) were used when lesions fell between any two categories. Lesion scores were compared using a Kruskal–Wallis test, with a Dunn’s post-hoc test for multiple pairwise comparisons.

### Intraperitoneal passive transfer and challenge of BALB/c mice

Thirty 8-week-old Female BALB/cJ mice were purchased from Jackson Laboratories (Jackson Laboratories, Bar Harbor ME) and designated to one of three groups. Each mouse received one IP injection containing 150 μL of either naïve mouse serum, LAMPs-induced serum, or dLAMPs-induced serum (pooled from donor mice) in 100 μL sterile physiologic saline, for a total volume of 250 μL. Twenty-four hours later, each mouse was inoculated intranasally with 50 μL containing 1 × 10^8^ CFU of *M. pneumoniae* PI1428 in FC media. Four days post challenge, all mice were euthanized, lung sections were taken for bacterial recovery, and then lungs were collected in 10% NBF for histologic analysis. All samples were treated as described above. Data analysis was conducted as described previously for IN serum passive transfers.

### Intranasal passive transfer of P1 monoclonal antibody and challenge in BALB/c mice

Twenty 8-week-old Female BALB/cJ mice were purchased from Jackson Laboratories (Jackson Laboratories, Bar Harbor ME) and separated into two groups assigned to receive one of the following treatments on a per animal basis; 1.76 μg of a-P1 monoclonal murine IgG1 antibody or 1.76 μg of murine IgG1 isotype control antibody. The mice were inoculated intranasally with 50 μL of Fc media containing both monoclonal antibody and live *M. pneumoniae* bacteria. The formulations provided to each animal at the time of infection contained 1.76 μg of their respective monoclonal antibody suspended in FC media containing 1 × 10^8^ live *M. pneumoniae* strain PI1428. Four days post inoculation, all animals were euthanized as described above.

### Intraperitoneal passive transfer of P1 monoclonal antibody and challenge in BALB/c mice

Thirty 8-week-old female BALB/cJ mice were purchased from Jackson Laboratories (Jackson Laboratories, Bar Harbor ME) and separated into three assigned to receive one of the following treatments; 6.44 μg of a-P1 monoclonal murine IgG1 antibody in 200 μL saline on study day −1, 6.44 μg of a-P1 monoclonal murine IgG1 antibody in 200 μL saline on study day +1, or 6.44 μg of murine IgG1 isotype control antibody in 200 μL saline on study day −1. All animals received administration of antibody via IP injection using a 25 G insulin syringe. On day 0 of the study, all thirty animals received an IN challenge with 1 × 10^8^ CFU *M. pneumoniae* strain PI1428 in 50 μL of Fc media. Four days post inoculation, all animals were euthanized by cervical dislocation under isoflurane anesthesia for sample collection. Lung samples were collected for bacterial recoveries and histopathologic analysis as described previously. All samples were processed and analyzed using the same protocols as described above.

### Vaccination with denatured dLAMPs and challenge in BALB/c mice

Sixty 8-week-old female BALB/cJ mice were split into two groups of *n* = 30. Each group was vaccinated on a prime-boost schedule on day 0 and day 21 with 50 μg of DdL or NdL in a total volume of 250 μL. Denatured dLAMPs were produced by incubation of NdL at 99 °C for 10 min in a thermocycler. Prior to inoculation of the animals, all vaccine formulations were mixed by briefly vortexing at maximum speed. On day 42 of the study, 20 animals from each group were challenged with 1 × 10^8^ CFU of *M. pneumoniae*. Four days post-challenge, all animals were euthanized by cervical dislocation under isoflurane anesthesia for sample collection. Samples for bacterial recovery and lung histology were collected as described above. Recoveries were calculated using the CCU method, and lesion scores were determined using the system described above. All data were analyzed using the Mann–Whitney U test. The remaining two groups of ten animals were sacrificed as described previously, and blood was immediately collected and stored as described above.

### Analysis of antibody responses to native and denatured dLAMPs by ELISA

Serum antibody responses in mice vaccinated with Native (NdL) or Denatured (DdL) dLAMPs were evaluated by ELISA. Nunclon Polysorp plates (Thermo Fisher Scientific, Waltham MA) were coated overnight at 4 °C with 100μL carb/bicarb buffer containing 5 μg/mL *M. pneumoniae* lysate. Prior to the loading of samples, plates were washed 3× with PBST containing 0.05% Tween-20 and blocked for 1 h with 100 uL of 2 μg/mL bovine serum albumin diluted in PBST. Plates were washed 3× with PBST, and 100 μL of diluted sample was loaded and incubated for 1 h at room temperature. Sample dilutions were as follows, based on sample source and antibody subtype/isotype queried: Serum IgG 1:10,000, Serum IgM 1:100, Serum IgG1 1:2000, Serum IgG2a 1:2000. Plates were then washed 3× and 100 μL of 1:4000 HRP-conjugated secondary antibody diluted in PBST was loaded and incubated for 1 h at room temperature. Plates were washed 3× with PBST and were developed and read as described above. Data points are the average of three technical replicates per animal.

### Vaccination, B cell depletion, and challenge of BALB/c mice

Twenty 8-week-old female BALB/cJ mice were purchased from Jackson Laboratories (Jackson Laboratories, Bar Harbor, ME) and into two groups. Three days prior to the beginning of the vaccination regimen (D-3), each group of mice received either 200 μg of mouse anti-CD20 clone 18B12.1 IgG2a or 200 μg of IgG2a isotype control in a 250 μL volume of physiological saline via IP injection. Three days later (D0) all mice were vaccinated with 50 μg of LAMPs in a 250 μL volume of physiological saline via IP injection. This process was then repeated with an additional administration of anti-CD20 or isotype antibody on day 18, followed by administration of a booster dose of LAMPs on day 21. On day 42, all mice were challenged via IN inoculation with 50 μL FC media containing 1 × 10^8^ CFU of *M. pneumoniae*. Four days post challenge (study day 46) all mice were euthanized, and samples were taken for bacterial recovery and lung histopathologic analysis as described above. All samples were processed and analyzed using the same protocols as described above.

### Histologic evaluation of mouse lungs after LAMPs-vaccination and B cell depletion

Histologic evaluation of the lung was performed in a blinded fashion. A scoring system was developed to include the presence, extent, and severity of pneumonia. Presence and extent were graded by percentage of tissue affected; 0, 1 (<20%), 2 (20–60%), and 3 (>60%). Severity scoring was a composite score of alveolar exudate (0–3), interstitial infiltrate and expansion (0–3), bronchiolar exudate (0–3), and alveolar edema (0–3). Since a range of lesion severity was present, for each component, a mean score was calculated by averaging the severity of four fields viewed at 40× objective. The severity of alveolar exudate and interstitial infiltration was determined by the cellularity of the inflammatory cells. For bronchiolar exudate, severity was determined by the number of bronchioles affected; 0.5 (1–5 bronchioles), 1 (5–10 bronchioles), and 1.5 (>10 bronchioles). For alveolar edema, severity was determined by the percentage of alveolar lumens affected in the field; 0, 1 (<20%), 2 (20–60%) and 3 (>60%). The presence of lymphoid aggregates (0–3) and neutrophilic aggregates (0–3) were scored separately. For lymphoid and neutrophilic aggregates, scoring was determined by the cellularity and arrangement of aggregates.

### RNAscope in situ hybridization

RNAscope in situ hybridization was conducted using the ACDbio RNAscope 2.5HD Duplex Manual Assay Kit (mouse) (Advanced Cell Diagnostics, Inc., Newark, CA) (Cat#322436) per manufacturer instructions for formalin-fixed paraffin-embedded tissues (as available at https://acdbio.com/technical-support/user-manuals). The probes used were: RNAscope 2.5HD Duplex Negative Control (Cat#320751), RNAscope 2.5HD Duplex Positive Control (Cat#321651), Mm-CD19-C1 (Cat#314711), Mm-CD4-C2 (Cat#406841-C2), Mm-IL-17A-C1 (Cat#319571). At the conclusion of the staining procedure, slides were stained using a 1:1 ratio of DI water and Gill’s Hematoxylin 1 (Fisher Scientific, Hampton, NH) (Ref#HXGHE1LT).

### Statistical analysis

All data analysis was done using GraphPad PRISM 8 (GraphPad Software, San Diego CA). Specific tests used for different data sets are described in the relevant methods sections as well as in each figure legend. Data were considered significant for *p* < 0.05 (*p < 0.05, **p < 0.01, ***p < 0.001).

### Reporting summary

Further information on research design is available in the Nature Research Reporting Summary linked to this article.

## Supplementary information

Supplemental Material

REPORTING SUMMARY

## Data Availability

The data supporting the findings described in this study are available within the paper and within the supplementary file.
