# Supplementary material for: B cells oppose Mycoplasma pneumoniae vaccine enhanced disease and limit bacterial colonization of the lungs
Source: NPJ Vaccines. 2022 Oct 31;7:130. doi: 10.1038/s41541-022-00556-z (PMC9618410; doi:10.1038/s41541-022-00556-z)
Supplement: Supplementary file 1 — Supplemental Material [file 41541_2022_556_MOESM1_ESM.pdf]

Supplementary Figures

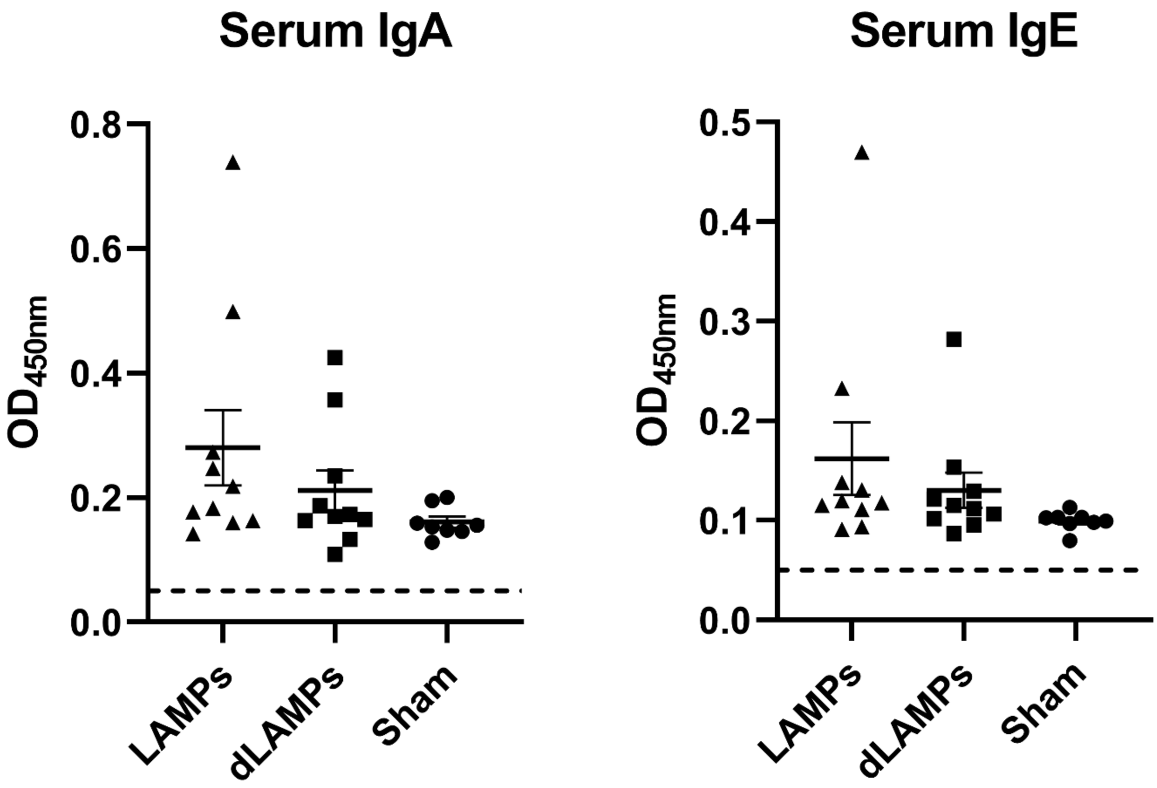

Supplementary Figure 1: Serum IgA and IgE titers in LAMPs, dLAMPs, and Sham-vaccinated mice. Serum was diluted 1:100 in PBST. Dotted line indicates lower limit of detection of the assay.

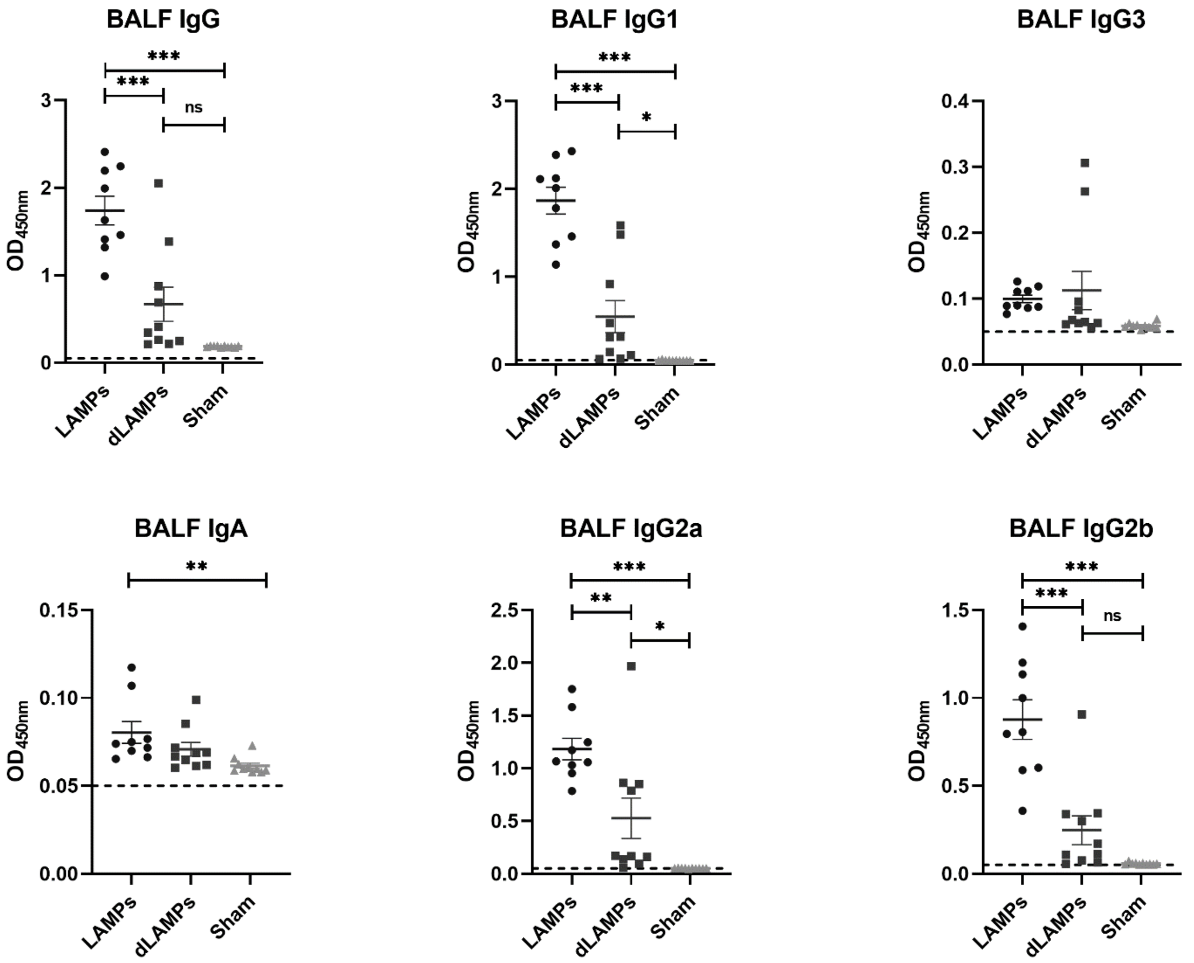

Supplementary Figure 2: Bronchoalveolar lavage fluid antibody responses to LAMPs and dLAMPs in vaccinated mice. BALF was diluted as follows: IgG 1:500, IgA 1:100, IgG1 1:100, IgG2a 1:100, IgG2b 1:100, IgG3 1:100. Dashed lines indicate the limit of detection of the Cytation 5 plate reader used to collect optical density values. Data were analyzed by One-Way ANOVA with Tukey's post-hoc test. Data was considered significant for $p<0.05$ (* $p<0.05$, ** $p<0.01$, *** $p<0.001$).

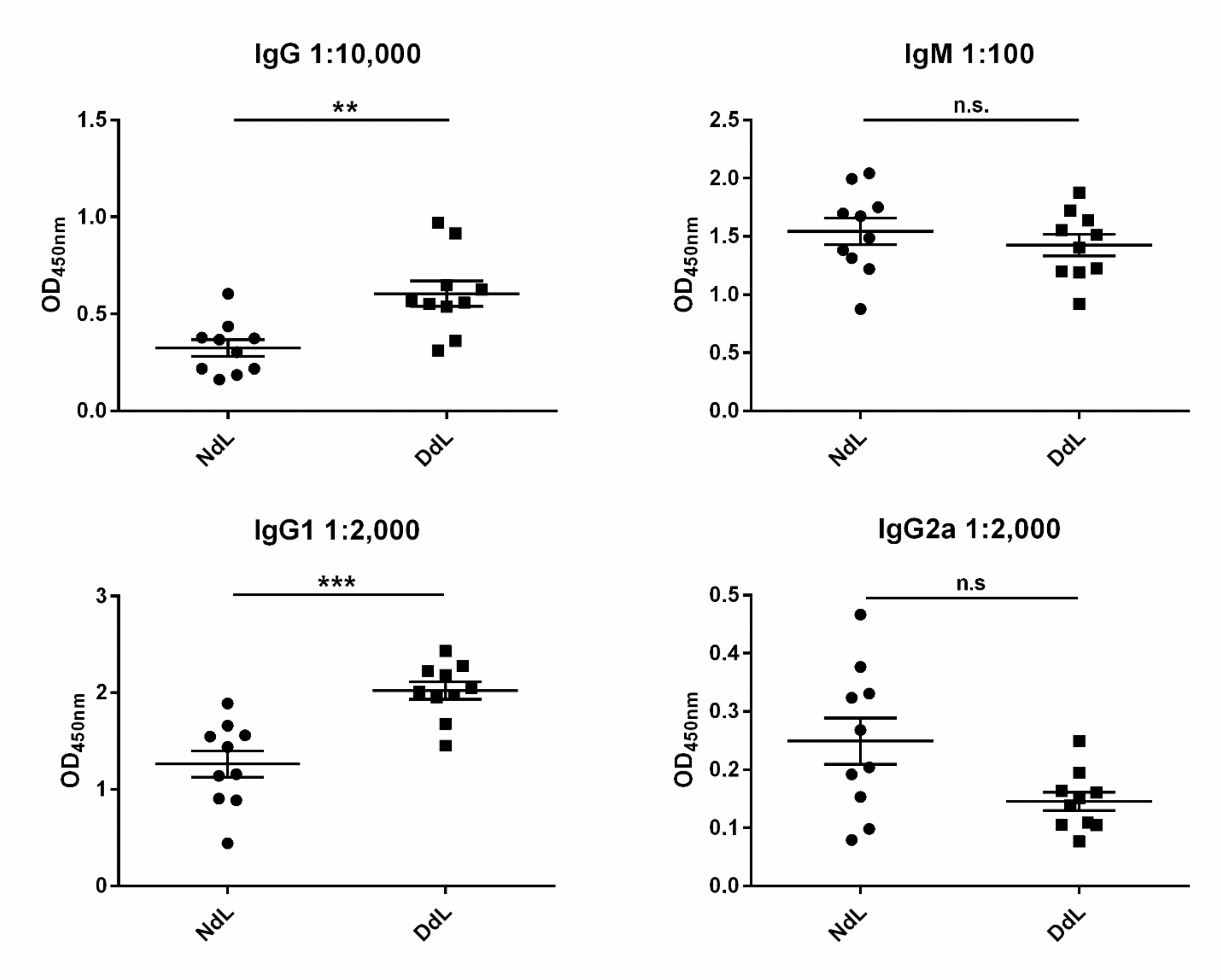

Supplementary Figure 3: Serum antibody responses to *M. pneumoniae* lysate in animals vaccinated with the native conformation dLAMPs fraction (NdL) or the heat denatured dLAMPs fraction (DdL). Data was analyzed using the Mann Whitney U test, and differences were considered significant for $p<0.05$ (* $p<0.05$, ** $p<0.01$, *** $p<0.001$).

.

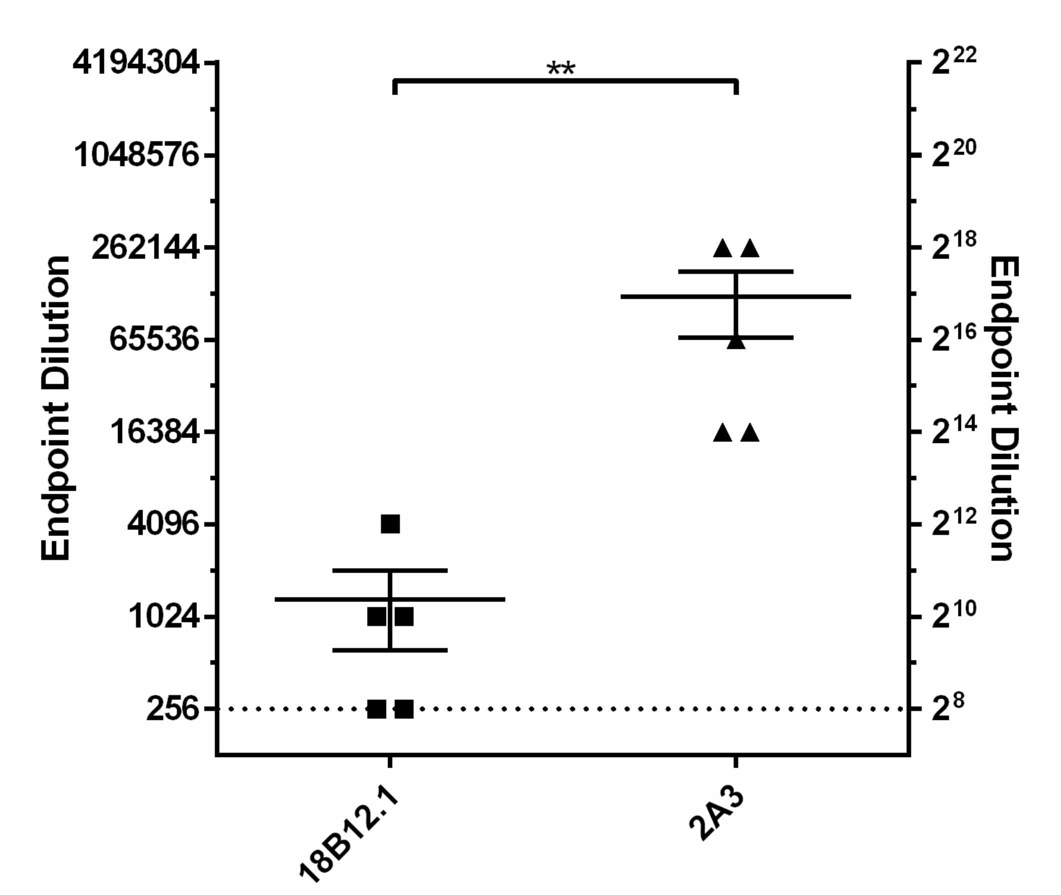

Supplementary Figure 4: Endpoint IgG antibody responses to *M. pneumoniae* lysate in animals administered a B cell depleting antibody (18B12.1) or isotype antibody (2A3) and then vaccinated with *M. pneumoniae* LAMPs. Sham animals received saline sham for both injections. Endpoint titers are displayed as the reciprocal of the final dilution at which the signal was at least 3 times greater than the assay background. Dotted line represents the average endpoint titer of sham-vaccinated mice. Data was analyzed by Mann-Whitney Test, with differences considered significant for $p<0.05$ (** $p<0.01$).

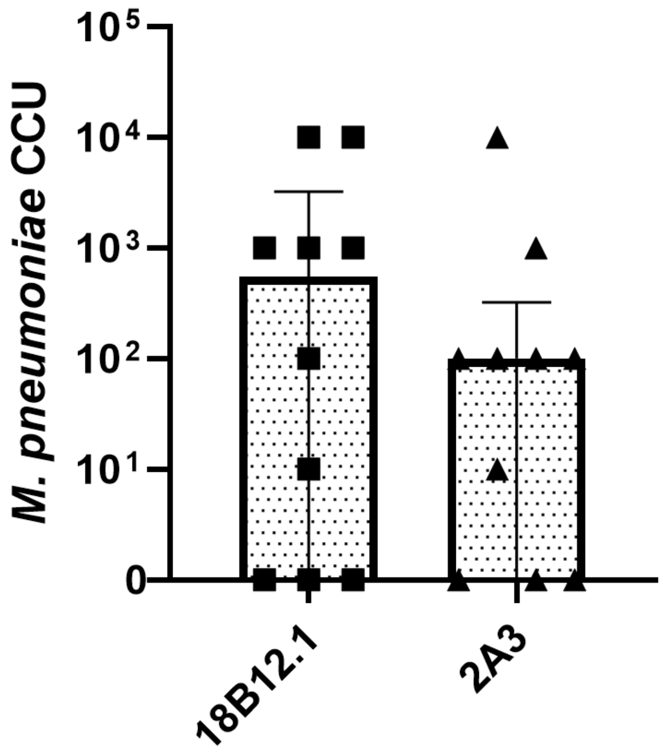

Supplementary Figure 5: Bacterial recoveries from the lungs of mice administered LAMPs-vaccination plus B cell depleting antibody (18B12.1) or plus isotype control antibody (2A3) and then challenged with *M. pneumoniae*. Data are median with interquartile range. Data was analyzed by Mann-Whitney Test.

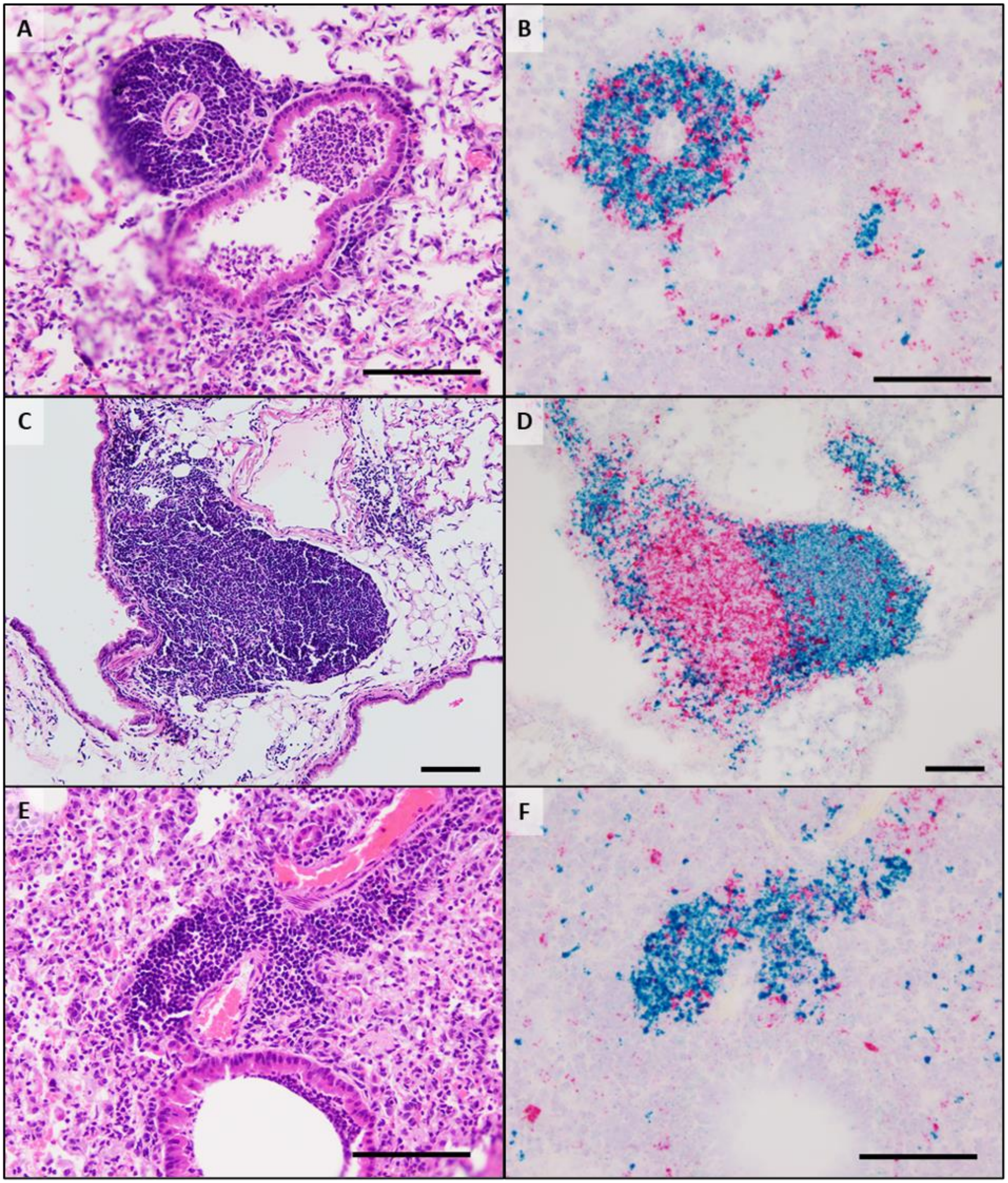
A
B
C
D
E
F

Supplementary Figure 6: H&E staining **[A/C/E]** and RNAscope in situ hybridization **[B/D/F]** of lung sections from mice vaccinated with the LAMPs fraction **[A/B]** or dLAMPs fraction **[C/D]** prior to challenge with live *M. pneumoniae*. Sham vaccinated and challenged animals were included as reference controls **[E/F]**. Blue chromogen deposits correspond to CD19 mRNA, red chromogen deposits correspond to CD4 mRNA. **A/B:** Pulmonary vessel and bronchiole in an area with alveolar and bronchiolar neutrophilic exudate. The perivascular cuff is composed mostly of cells with CD19mRNA. There is a relatively smaller peribronchiolar cuff with circumferentially arranged cells with CD4 mRNA and multifocal aggregates of cells with CD19mRNA. **C/D:** Between a bronchiole and vessel there is a large lymphoid aggregate (iBALT) in a dLAMPs vaccinated mouse. RNAscope in situ hybridization of this lesion has two clearly defined zones of cells with CD19 mRNA and cells with CD4 mRNA. **E/F:** In a Sham vaccinated mouse, the alveolar septa are markedly expanded by high numbers of macrophages accompanied by a neutrophilic exudate within the lumen of bronchioles and alveoli. Within this area there is a perivascular cuff with RNAscope in situ hybridization showing a distribution of high numbers of cells with CD19 mRNA and lesser numbers of cells with CD4 mRNA. All scale bars are equivalent to 100μm.

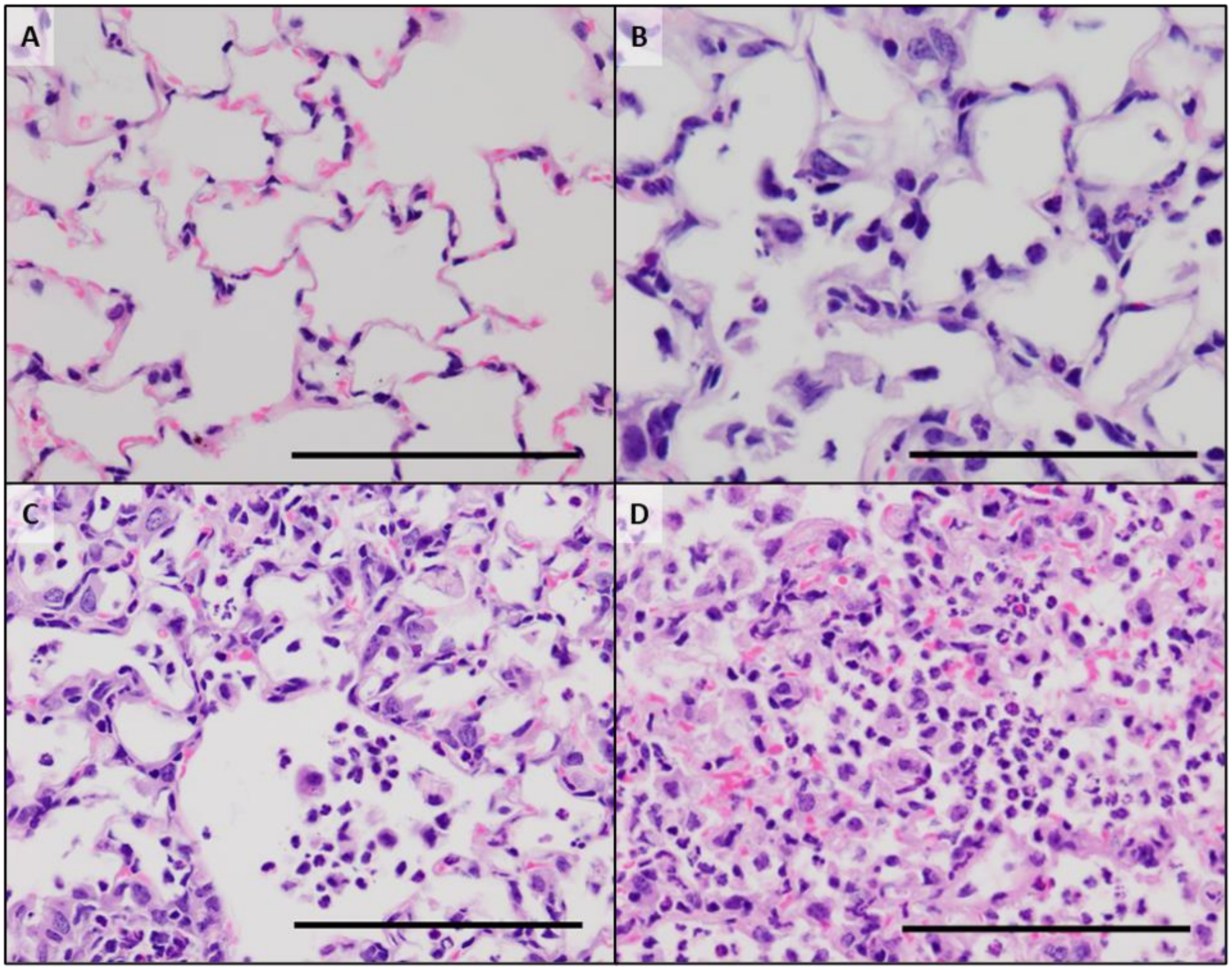

Supplementary Figure 7: Representative H&E-stained histologic sections correlating to the severity scores utilized in the B cell depletion study. **A.** Severity score 0. Representative region of normal lung. **B.** Severity score 1. A low number of alveoli contain individual to small aggregates of neutrophils and occasional macrophages within their lumen. Alveolar septa are multifocally expanded by low numbers of lymphocytes and macrophages. **C.** Severity score 2. Most alveolar lumens contain relatively low numbers of neutrophils and macrophages, and the septa are frequently expanded by lymphocytes and macrophages. **D.** Severity score 3. High numbers of neutrophils and lesser numbers of macrophages fill the alveolar lumen mixed with protein-rich edema, with little remaining air space. Alveolar septa are diffusely expanded by mononuclear cells. H&E. Scale bar = 100μm.
